# A novel heterozygous variant of the *COL4A4* gene in a Chinese family with hematuria and proteinuria leads to focal segmental glomerulosclerosis and chronic kidney disease

**DOI:** 10.1002/mgg3.1545

**Published:** 2020-11-07

**Authors:** Liang‐Liang Fan, Lv Liu, Fang‐Mei Luo, Ran Du, Chen‐Yu Wang, Yi Dong, Ji‐Shi Liu

**Affiliations:** ^1^ Department of Nephrology The Third Xiangya Hospital of Central South University Changsha China; ^2^ Department of Cell Biology The School of Life Sciences Central South University Changsha China; ^3^ Hunan Key Laboratory of Animal for Human Disease School of Life Sciences Central South University Changsha China; ^4^ Department of Respiratory Medicine, Diagnosis and Treatment Center of Respiratory Disease the Second Xiangya Hospital of Central South University Changsha Hunan China

**Keywords:** COL4A4 mutation, focal segmental glomerulosclerosis, hematuria, proteinuria

## Abstract

**Background:**

Focal segmental glomerulosclerosis (FSGS), as the frequent primary glomerular diseases in adults, accounts for symptomless proteinuria or nephrotic syndrome with or without renal insufficiency. As the crucial lesion of chronic kidney disease (CKD), accumulating evidence from recent studies show that mutations in Collagen‐related genes may be responsible for FSGS. The aim of this study was to identify the genetic lesion of a Chinese family with FSGS and CKD.

**Methods:**

In this study, we recruited a Han‐Chinese family with unexplained high serum creatinine, hematuria, and proteinuria. Further renal biopsy and renal pathology indicated the diagnosis of FSGS in the proband. Whole‐exome sequencing and Sanger sequencing were employed to explore the pathogenic mutation of this family.

**Results:**

A novel heterozygous mutation (NM_000092 c.2030G>A, p.G677D) of the *collagen type IV alpha*‐*4* gene (*COL4A4*) was detected. Co‐segregation analysis revealed that the novel mutation was carried by all the five affected individuals and absent in other healthy members as well as in our 200 local control cohorts. Bioinformatics predication indicated that this novel mutation was pathogenic and may disrupt the structure and function of type IV collagen. Simultaneously, this variant is located in an evolutionarily conserved site of COL4A4 protein.

**Conclusion:**

Here, we identified a novel mutation of *COL4A4* in a family with FSGS and CKD. Our study expanded the variants spectrum of the *COL4A4* gene and contributed to the genetic counseling and prenatal genetic diagnosis of the family. In addition, we also recommended the new classification of collagen IV nephropathies, which may be a benefit to the diagnosis, target drug treatment, and management of patients with *COL4A3*/*COL4A4* mutations.

## Graphical abstract

This study identified a novel heterozygous mutation (NM_000092 c.2030G>A, p.G677D) of the collagen type IV alpha‐4 gene (COL4A4), which may broaden the phenotypic spectrum of type IV collagen nephropathies. In addition, together with previous studies, we also recommended the new classification of collagen IV nephropathies, which may be a benefit to the diagnosis, target drug treatment, and management of patients with COL4A3/COL4A4 mutations.

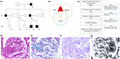

## 1 INTRODUCTION

Focal segmental glomerulosclerosis (FSGS), the major cause of primary glomerular diseases and chronic kidney disease (CKD), is characterized by scar tissue that forms in some of the glomeruli in the kidney (D'Agati et al., 2011; Rosenberg & Kopp, 2017). As a progressive form of kidney disease, FSGS results in symptomless proteinuria or nephrotic syndrome with or without renal insufficiency and accounts for 2.3% of end‐stage renal disease (Laurin et al., 2016). At present, FSGS is divided into idiopathic FSGS, secondary FSGS, and genetic FSGS (D'Agati et al., 2011).

FSGS results from a variety of systemic disorders, such as obesity, sickle cell disease, and HIV infection. In addition, a rare form of FSGS depends on genetic factors (Fogo, 2015; Lim et al., 2016). Currently, approximately 20 pathogenic genes involved in the maintenance of podocyte structure and function have been identified in FSGS patients (Bose et al., 2014; J. Liu & Wang, 2017). According to the inherited patterns, these genes can be classified into three groups: (i) autosomal dominant inheritance, for example, *PAX2* (OMIM# 167409), *ANLN* (OMIM# 616027), *COL4A3* (OMIM# 120070), *COL4A4* (OMIM# 120131), *COL4A5* (OMIM# 303630), *CD2AP* (OMIM# 604241), *ARHGAP24* (OMIM# 610586), *TRPC6* (OMIM# 603652), *INF2* (OMIM# 610982), *ACTN4* (OMIM# 604638), and *LMX1B* (OMIM# 602575); (ii) autosomal recessive form, such as *NPHS1* (OMIM# 602716), *NPHS2* (OMIM# 604766), *ITGB4* (OMIM# 147557) and *TTC21B* (OMIM# 612014); (iii) X‐linked recessive model, for example, *NXF5* (OMIM# 300319).

The *COL4A4* gene (OMIM 120131) locates in the 2q36.3 and encodes one of the six subunits of type IV collagen, the major structural composition of basement membranes (Cosgrove & Liu, 2017). In 1994, two mutations of *COL4A4* were detected in two families with autosomal recessive Alport syndrome (AS) (Mochizuki et al., 1994). Since then, more than 200 *COL4A4* mutations have been described in AS sufferers. However, there were also several studies have detected the mutations of *COL4A4* in the families with hereditary FSGS, which indicated that the glomerular basement membrane (GBM) lesions caused by *COL4A4* mutations were responsible for hereditary FSGS (Malone et al., 2014; Pierides et al., 2009; Voskarides et al., 2007; Wu et al., 2016).

Here, we enrolled in a Han‐Chinese family with unexplained high serum creatinine, hematuria, and proteinuria. Further renal biopsy and renal pathology confirmed the diagnosis of FSGS in the proband. What is more, a novel heterozygous mutation (NM_000092 c.2030G>A, p.G677D) of the COL4A4 gene was confirmed to be genetic lesion of this family by employed whole‐exome sequencing (WES) and Sanger sequencing.

## 2 METHODS

### 2.1 Ethical compliance

This study was approved by the Ethics Committee of the Third Xiangya Hospital of Central South University, Changsha, China and performed in accordance with the principles enshrined in the Declaration of Helsinki. The patients/participants provided their written informed consent to participate in this study.

### 2.2 Subjects

**FIGURE 1:**
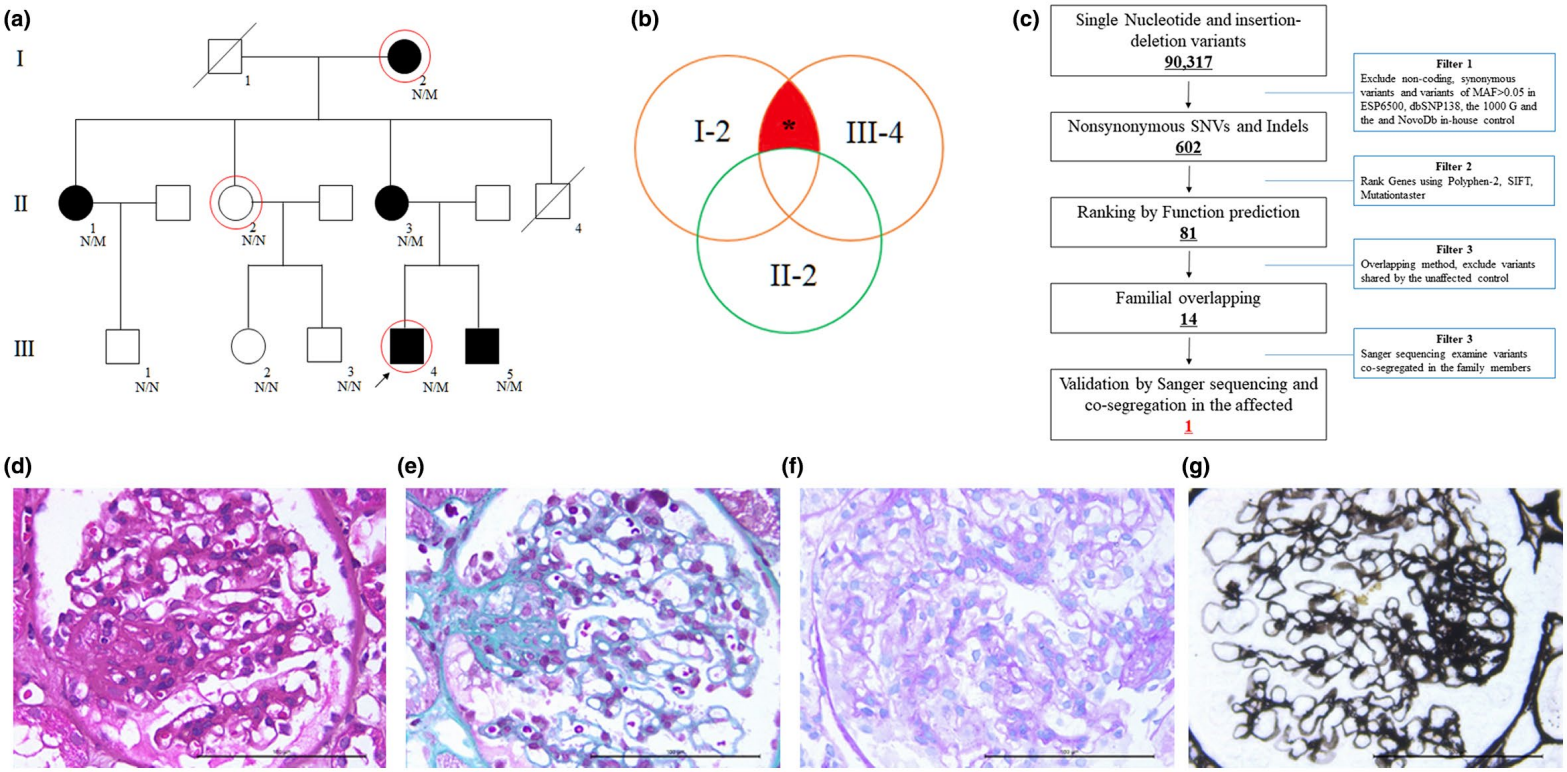
The clinical data of the family with FSGS. (a) Pedigree of the family with FSGS. Black circles/squares are affected, white are unaffected. Arrow indicates the proband. N means normal; M shows the *COL4A4* c.2030G>A variant. Three large circles in red represent the three individuals underwent whole‐exome sequencing. (b) Overlapping filter strategy. Asterisks denote remaining variants for further analysis that are present in two affected members (I‐2 and III‐4) but not in the normal control (II‐2). (c) Schematic representation of the filter strategies employed in this study. (d) HE staining (e) Masson staining (f) PAS staining and (g) PASM staining for renal biopsy of the proband (III‐4)

The family including 12 persons was investigated in this study (Figure 1a). The peripheral blood samples of five patients (I‐2, II‐1, II‐3, III‐4, and III‐5) and four healthy people (II‐2, III‐1, III‐2, and III‐3) were collected and applied to isolate Genomic DNA by Universal Genomic DNA Extraction Kit (Solarbio, D2100) as we have described (J. S. Liu et al., 2017). Simultaneously, clinical data such as renal function, urine testing, liver function, etc., were recorded carefully. The renal biopsy and renal pathology of the proband (III‐4) were performed by Masson, Periodic Acid‐Schiff (PAS), and Periodic Acid‐Silver Methylamine (PASM) staining. In addition, 200 unrelated local healthy people were also enrolled to serve as normal controls.

### 2.3 Whole‐exome sequencing

Two affected members (III‐4 and I‐2) and one healthy people (II‐2) were selected to perform whole‐exome sequencing (Figure 1b). Exome capture and next‐generation sequencing were conducted by Novogene Bioinformatics Institute. One microgram of qualified genomic DNA from each person was captured by the Agilent's SureSelect Human All Exon kit V5 (Agilent Technologies, Inc.) and sequenced by Illumina HiSeq 4000 (Illumina Inc.). Shortly, genomic DNA was randomly carved by Covaris S220 sonicator (Covaris, Inc.) (Fan, et al., 2019). Then the fragmented DNAs underwent three enzymatic steps: end repair, A‐tailing, and adapters ligation. The adapter‐ligated DNA fragments were amplified with Herculase II Fusion DNA Polymerase (Agilent). Finally, the exomes in the pre‐capture libraries were captured by the SureSelect capture library kit (Agilent) (Fan, et al., 2019). After DNA quality assessment, the captured DNA library was used for next‐generation sequencing on Illumina HiSeq 4000 platform (Fan, et al., 2019). Downstream processing was carried out by Genome Analysis Toolkit (GATK), Varscan2, and Picard, and variant calls were made with the GATK Haplotype Caller (Fan, et al., 2019). Variant annotation referred to Ensemble release 82, and filtering was conducted by ANNOVAR Documentation.

Non‐synonymous SNPs or frameshift‐causing INDELs with an alternative allele frequency >0.005 in the NHLBI Exome Sequencing Project Exome Variant Server (ESP6500), dbSNP147 (http://www.ncbi.nlm.nih.gov/projects/SNP/index.html), the 1000 Genomes project (http://www.1000genomes.org/), the ExAC database (http://exac.broadinstitute.org) or in‐house exome databases of Novogene (2500 exomes) were kicked before further analysis (Fan, et al., 2019). Then the filtered SNVs and INDELs, predicted by SIFT (http://sift.jcvi.org/), Polyphen2 (http://genetics.bwh.harvard.edu/pph2/) and MutationTaster (http://www.mutationtaster.org/) to be damaging, were remained (Liu et al., 2017). Finally, mutations exist in two affected members (I‐2 and III‐4), but absent in the healthy individual (II‐2) were withheld (Figure 1c).

### 2.4 Variant validation and co‐segregation analysis

Co‐segregation analysis was performed on each member by Sanger sequencing based on the aforementioned remaining variants after data filtering (Fan, et al., 2019). Simultaneously, the candidate variants also need to be excluded the possibility of local polymorphism in our 200 unrelated local healthy people by Sanger sequencing (J. S. Liu et al., 2017).

## 3 RESULTS

The proband (III‐4), a 15‐year‐old male, came to our hospital due to the abnormal urine test before university admission. The chief complaint was felled dizzy in recent one month. Basic testing found that patients presented high blood pressure (164/102 mmHg), with mild swelling of the face and lower extremities. No discomfort such as hair loss, rash, abdominal pain, diarrhea, joint pain. Further examinations were shown as follow (Table 1): urine testing: proteinuria 2+, hematuria 1+; renal function: blood urea nitrogen 8.37 mmol/L, blood creatinine 144 μmol/L, uric acid 497.5 μmol/L. Other medical testing including liver function, blood lipid, blood glucose, erythrocyte sedimentation rate, connective tissue, and vasculitis‐related tests, immunity, hepatitis and HIV antibody tests, tumor markers, etc., which did not display significant abnormality. Kidney size is normal. Diabetes, hepatitis‐related nephropathy, lupus nephritis, obstructive kidney disease, and tumor‐related kidney disease are excluded. Further renal biopsy and renal pathology testing showed diffuse increased glomerular volume, focal segmental mesangial hyperplasia, endothelial cell vacuole degeneration, small focal foot adenopathy hyperplasia, segmental sclerosis and adhesion to the balloon, narrowing of the renal balloon, no new Lunar formation (Figure 1d). Masson, PAS, and PASM staining mesangial area showing mesophilic deposition (Figure 1e–g). Renal biopsy and renal pathology testing are consistent with the diagnosis of FSGS. Family history investigation found that his grandmother (I‐2), mother (II‐3), one aunt (II‐1), and one brother (III‐5) all suffered from hematuria and proteinuria for more than 3 months (Table 1).

**Table 1:** Clinical and genetic data of five patients with *COL4A4* c.2030G>A (p.G677D) variation

Subjects	III‐4 (proband)	I‐2	II‐1	II‐3	III‐5	Normal
Sex	M	F	F	F	M	/
Age (years)	20	72	49	43	16	/
Genotype	Heterozygote	Heterozygote	Heterozygote	Heterozygote	Heterozygote	/
Microscopic hematuria	1+	1+	1+	1+	1+	—
Proteinuria	2+	3+	2+	2+	1+	—
Uraemia	No	No	No	No	No	/
Blood creatinine (μmol/L)	144	220	214.5	202.8	134.4	M: <106; F: <86
Blood urea nitrogen (mmol/L)	8.37	12.82	11.03	12.58	8.08	1.8‐7.1
Uric acid (μmol/L)	497.5	608.9	569	521.4	457.3	M: 149–416; F: 89–357
Audiological examination	Normal	Normal	Normal	Normal	Normal	/
Ophthalmic examination	Normal	Normal	Normal	Normal	Normal	/
BMI (kg /m^2^)	21.9	22.7	22.5	23	21.8	18.5–24.9

Abbreviations: BMI, body mass index; F, female; M, male.

**FIGURE 2:**
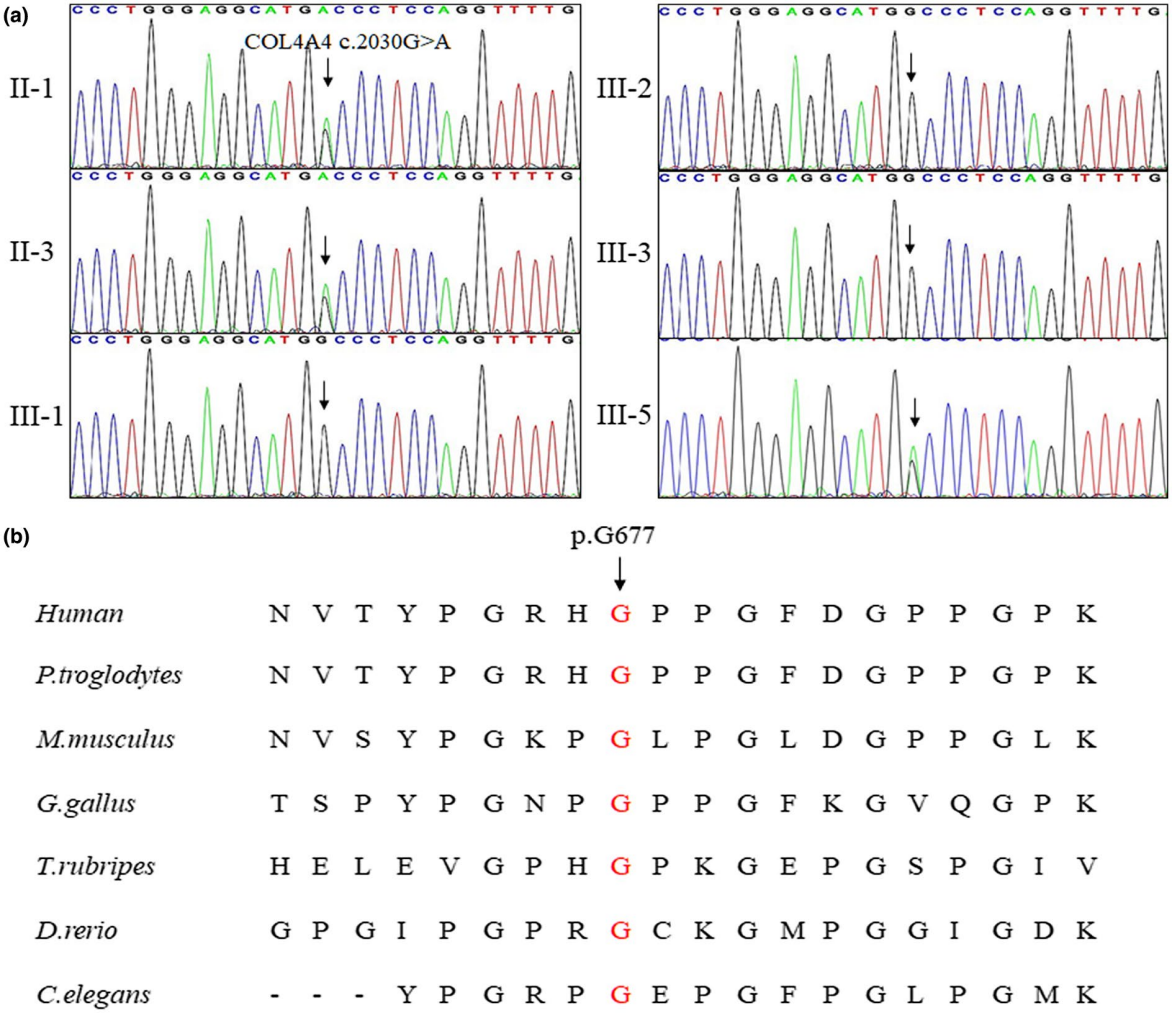
The Sanger sequencing and conservation analysis of the novel variant. (a) Sanger DNA sequencing chromatogram demonstrates the heterozygosity for a *COL4A4* missense mutation (*COL4A4* c.2030G>A, p.G677D) in affected members. (b) Alignment of multiple COL4A4 protein sequences across species. The affected G677 amino acid is located in a highly conserved region in different mammals (from Ensembl). The letter in red shows the G677 site

The 99.8, 99.6, and 99.8% coverage of the target regions, and 130.82×, 101.60×, and 175.40× sequencing depth were achieved for III‐4, I‐2, and II‐2, respectively. Totally about 90,317 variants were detected in the proband (III‐4) (Table S1). Via abovementioned filtering method and familial overlapping (Figure 1c), a novel heterozygous mutation (NM_000092 c.2030G>A, p.G677D, RNA not analyzed) of *COL4A4* gene was present in the two patients (I‐2 and III‐4), but absent in the unaffected individual (II‐2). No other potential pathogenic mutations for kidney diseases were found (Table S2). Sanger sequencing further confirmed that this novel mutation (NM_000092 c.2030G>A, p.G677D) of *COL4A4* was present in another three affected family members (II‐1, II‐3, and III‐5), while absent in three uninfluenced individuals (III‐1, III‐2, and III‐3) (Figure 2a). The novel mutation, generating a substitution of glycine by aspartic acid in exon 26 of the *COL4A4* gene, was devoid in our 200 local control cohorts. Bioinformatics methods predicated that this mutation (NM_000092 c.2030G>A, p.G677D) was a pathogenic mutation and located in an evolutionarily conserved site of COL4A4 protein highly evolutionarily conserved (Figure 2b). According to ACMG guidelines (Richards et al., 2015), this mutation belongs to PM1+PM2+PP3+PP1.

## 4 DISCUSSION

The typical clinical presenting symptoms of FSGS is nephrotic syndrome, marked by generalized edema, massive proteinuria, hypoalbuminemia, and hyperlipidemia (Bose et al., 2014; D'Agati et al., 2011; Nagata et al., 2017). Previous studies demonstrated that 10%–38% familiar FSGS were caused by mutations in *COL4A3–5* which encoded the type IV collagen (De Vriese et al., 2018; Liu & Wang, 2017). Given that the candidate genes of FSGS are many and the causative *COL4A3–5* genes are large, large‐scale FSGS‐related genetic analyses by traditional Sanger sequencing is a waste of time and money. WES is a presently efficient way to detect genetic lesions of familiar FSGS (Lin et al., 2014). A novel heterozygous mutation (NM_000092 c.2030G>A, p.G677D) of the *COL4A4* gene was verified in a Han‐Chinese FSGS pedigree via WES. The new mutation co‐segregates sharply with the FSGS phenotypes, which was confirmed by renal biopsy and renal pathology testing. Our study in accordance with formerly research indicated that variants in *COL4A4* can disrupt the structure and function of GBM and finally lead to hereditary FSGS (Papazachariou et al., 2014) and CKD.

The human *COL4A4* gene locates at chromosome 2q36.3 and spans approximately 161 kb and consists of 48 exons. The *COL4A4* is organized in a head‐to‐head conformation with *COL4A3*, hence both genes share one promoter to encode different chains of type IV collagen, respectively (Gale et al., 2016). Both *COL4A3* and *COL4A4* are specifically expressed in the GBM, inner ear, and eye (Nogueira et al., 2000). The pathomechanism between abnormal GBM and FSGS has not been fully understood currently, while several studies considered the damaging of podocytes, and the adherences between the parietal epithelial cells and naked GBM as the crucial process in FSGS (Campbell & Tumlin, 2018). The most recent study employed single‐cell sequencing in FSGS patients’ samples and found that the activation of glomerular endothelial cells was overt in immunosuppressive naive patients with FSGS (Menon et al., 2020). Another study in the mouse model revealed that genes encoding podocyte cytoskeletons were essential for FSGS (Lu et al., 2017). The COL4A4 protein was produced from podocytes and plays a crucial role in GBM (Sado et al., 1998). The abnormal collagen IV a3/a4/a5 chains can impair the GBM and induce the abnormal signal to affect the adherent endothelial cells and podocytes, finally result in FSGS (Abrahamson, 2012). Here, we identified a novel *COL4A4* mutation in a Han‐Chinese pedigree with FSGS and CKD. Further functional study such as single‐cell RNA‐seq may provide a new sight in the pathomechanism between GBM and FSGS.

The human COL4A4 protein consists of a 25‐amino acid N‐terminal domain, the central triple‐helical domain including G‐X‐Y repeats and the C‐terminal globular noncollagenous domain (Longo et al., 2002; Sado et al., 1998; Storey et al., 2013). Previous studies have proved that pathogenic mutations in G‐X‐Y repeats may affect the flexibility of the central triple‐helical domain and produce abnormal α4 chains of type IV collagen, which may disrupt the stability of THE molecular superstructure of collagen (Sado et al., 1998). Here, the newly identified mutation was seated in the G‐X‐Y repeats and led to the substitution of glycine by aspartic acid, which may disrupt the flexibility and stability of type IV collagen, finally impair the structure and function of GBM and induce FSGS (Longo et al., 2002; Sado et al., 1998; Storey et al., 2013). Glycine substitution mutations were the most common variant pattern in the *COL4A4* gene (Longo et al., 2002; Papazachariou et al., 2014). In our study, all the heterozygous glycine substitution mutation (NM_000092 c.2030G>A, p.G677D) carriers only presented FSGS phenotypes, none of our family members developed to end‐stage renal disease (ESRD), which indicates that glycine substitution mutations are always leading to milder symptoms, rather than more serious defects such as deletions/insertions or frameshift mutations in *COL4A4* gene. Moreover, four of five family members in this study are young‐onset and display clinical manifestations of proteinuria and renal dysfunction, indicating that the glycine substitution mutations may cause rapid progression of CKD and high risk of ESRD at later ages (Deltas et al., 2015; Pescucci et al., 2004; Voskarides et al., 2007).

According to previous studies, heterozygous *COL4A3*/*COL4A4* mutations can lead to AS, thin basement membrane disease, segmental GBM thin, as well as FSGS (Lin et al., 2014; Lu et al., 2017; Malone et al., 2014; Storey et al., 2013; Zhu et al., 2018). Because FSGS can be a secondary risk factor to AS and thin basement membrane disease, FSGS may be a process in the development of AS and GBM related diseases caused by variants in *COL4A3*/*COL4A4* (Zhu et al., 2018). Given the same genetic lesion, the *COL4A3*/*COL4A4* related FSGS, AS and GBM related diseases should be classified as subtypes of collagen IV nephropathies, which have been encouraged in previous reports (Wu et al., 2016). In this study, we also support this point of view. And this classification of collagen IV nephropathies may also contribute to the diagnosis and target drug treatment, as well as the management of this disorder.

Here, we reported a novel heterozygous mutation (NM_000092 c.2030G>A, p.G677D) of the *COL4A4* gene in a Han‐Chinese family with unexplained high serum creatinine, hematuria, and proteinuria. Renal biopsy and pathological testing confirmed the diagnosis of FSGS in the proband. The identification of this *COL4A4* c.2030G>A mutation may contribute to genetic counseling and prenatal genetic diagnosis of the FSGS patients, especially for the family members III‐3 and III‐4, two unmarried people, to reduce familial transmission in this family. Additional functional analysis of the COL4A4 protein with this mutation is recommended and may discover additional information about the pathogenetic mechanism of FSGS (Sado et al., 1998).

In summary, by employing WES and Sanger sequencing, a novel heterozygous mutation (NM_000092 c.2030G>A, p.G677D) of the *COL4A4* gene was detected in a Han‐Chinese pedigree diagnosed as hereditary FSGS and CKD. Hence, our study not only expanded the variants spectrum of *COL4A4* gene and contributed to the genetic counseling and prenatal genetic diagnosis of the family, but also recommended the new classification of collagen IV nephropathies, which may be a benefit to the diagnosis, target drug treatment, and management of *COL4A3*/*COL4A4* related FSGS, AS and GBM related diseases.

## CONFLICT OF INTEREST

The authors declare that they have no competing interests.

## AUTHORS’ CONTRIBUTIONS

Liang‐Liang Fan and Liu Lv enrolled the samples; Liang‐Liang Fan performed renal biopsy and renal pathology testing, whole‐exome sequencing and bioinformatics analysis; Lv Liu revised the manuscript; Fang‐Mei Luo and Ran Du isolated the gDNA; Chen‐Yu Wang and Yi Dong performed PCR and Sanger sequencing. Ji‐Shi Liu designed the project and supported it. Liang‐Liang Fan, Lv Liu and Ji‐Shi Liu wrote the manuscript. All the authors reviewed the manuscript.

## Supporting information

Table S1‐S2
