# Supplementary material for: A novel heterozygous variant of the COL4A4 gene in a Chinese family with hematuria and proteinuria leads to focal segmental glomerulosclerosis and chronic kidney disease
Source: Mol Genet Genomic Med. 2020 Nov 7;8(12):e1545. doi: 10.1002/mgg3.1545 (PMC7767549; doi:10.1002/mgg3.1545)
Supplement: Supplementary file 1 — Table S1‐S2 [file MGG3-8-e1545-s001.docx]

Table S1. Quality control data of whole exome sequencing

| Subjects | III-4 | I-2 | II-2 |
| --- | --- | --- | --- |
| Raw data yield (Mb) | 10786 | 8269 | 15240 |
| Target region (bp) | 39M | 39M | 39M |
| Coverage of target region (%) | 99.83 | 99.64 | 99.86 |
| Average sequencing depth | 130.82 | 101.60 | 175.40 |
| Fraction of target covered >=10X (%) | 99.45 | 99.22 | 99.56 |
| Fraction of target covered >=20X (%) | 99.02 | 98.61 | 99.25 |
| Fraction of target covered >=30X (%) | 98.34 | 97.46 | 98.85 |
| Total number of SNVs | 90317 | 101750 | 102985 |

Table S2. The data after whole exome sequencing filtering

| Gene | Transcript variant | Protein variant | OMIM |
| --- | --- | --- | --- |
| COL4A4 | NM_000092:c.2030G>A | p.G677D | Familial hematuria; AD |
| ABCA7 | NM_019112:c.1374delC | p.G458GfsX36 | Alzheimer disease; AD |
| DNAJC21 | NM_194283:c.706G>A | p.E236K | Bone marrow failure syndrome; AR |
| EGFR | NM_005228:c.1880+3G>A | - | Nonsmall cell lung cance; AD |
| KRT3 | NM_057088:c.1505_c.1508delACCG | p.Y502SfsX48 | Meesmann corneal dystrophy |
| GPR98 | NM_032119:c.10594T>C | p.W3532R | Usher syndrome; AR |
| KIF7 | NM_198525:c.3964T>C | p.S1322P | Joubert syndrome; AR |
|  | NM_198525:c.1071G>A | p.W357X | Joubert syndrome; AR |
| MEFV | NM_000243:c.2229C>G | p.F743L | Familial Mediterranean fever; AD/AR |
| POLG | NM_002693:c.2890C>T | p.R964C | Progressive external ophthalmoplegia; AD  Mitochondrial recessive ataxia syndrome; AR |
| RYR1 | NM_000540:c.11518G>A | p.V3840I | Neuromuscular disease; AD/AR |
| SLC36A2 | NM_181776:c.655C>T | p.R219W | Hyperglycinuria; AD |
| TIMMDC1 | NM_016589:c.652C>T | p.Q218X | Mitochondrial complex I deficiency; AR |
| XPNPEP2 | NM_003399:c.1183_c.1184delTT | p.F395Lfs*22 | Susceptibility to angioedema induced by ACE inhibitors |

AD, autosomal dominant; AR, autosomal recessive.
